# 3D-Printed Architected Materials Inspired by Cubic Bravais Lattices

**DOI:** 10.1021/acsbiomaterials.0c01708

**Published:** 2021-07-26

**Authors:** Flavia Libonati, Serena Graziosi, Federico Ballo, Marco Mognato, Giacomo Sala

**Affiliations:** † Department of Mechanical, Energy, Management and Transportation Engineering (DIME) Polytechnic School,University of Genoa, Via all’Opera Pia 15/A, Genova 16145, Italy; ‡ Department of Mechanical Engineering, Politecnico di Milano via La Masa 1, Milano 20156, Italy

**Keywords:** bioinspired materials, trabecular structure, lattice structure, 3D printing, lightweight structure

## Abstract

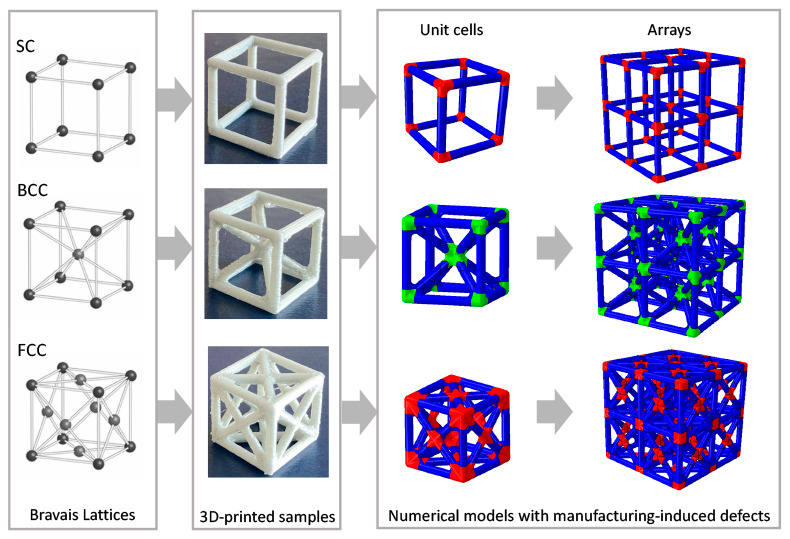

Learning from Nature and leveraging 3D printing, mechanical testing, and numerical modeling, this study aims to provide a deeper understanding of the structure–property relationship of crystal-lattice-inspired materials, starting from the study of single unit cells inspired by the cubic Bravais crystal lattices. In particular, here we study the simple cubic (SC), body-centered cubic (BCC), and face-centered cubic (FCC) lattices. Mechanical testing of 3D-printed structures is used to investigate the influence of different printing parameters. Numerical models, validated based on experimental testing carried out on single unit cells and embedding manufacturing-induced defects, are used to derive the scaling laws for each studied topology, thus providing guidelines for materials selection and design, and the basis for future homogenization and optimization studies. We observe no clear effect of the layer thickness on the mechanical properties of both bulk material and lattice structures. Instead, the printing direction effect, negligible in solid samples, becomes relevant in lattice structures, yielding different stiffnesses of struts and nodes. This phenomenon is accounted for in the proposed simulation framework. The numerical models of large arrays, used to define the scaling laws, suggest that the chosen topologies have a mainly stretching-dominated behavior—a hallmark of structurally efficient structures—where the modulus scales linearly with the relative density. By looking ahead, mimicking the characteristic microscale structure of crystalline materials will allow replicating the typical behavior of crystals at a larger scale, combining the hardening traits of metallurgy with the characteristic behavior of polymers and the advantage of lightweight architected structures, leading to novel materials with multiple functions.

## 1 Introduction

A long-sought goal of engineering design is to pursue lightweight structural materials with optimal strength-to-weight and stiffness-to-weight performance. Nature offers several effective solutions, especially for porous structures, from shock-absorbing hedgehog’s spines to trabecular bone. The former allows the hedgehog to bounce when it falls from a height, thus preventing injuries without overloading the animal. This structure, similar to a foam that fills the central part of a spine, supports the thin outer wall, contrasting local instability and allowing the whole system to bend further without breaking.^1,2^ The latter (trabecular bone, aka spongy or cancellous bone) is another classic example of cellular structure at the microscale. It has an open-cell porous arrangement, apparently random, but carefully designed by Nature to bear specific local loadings^3^ and to fulfill different functional needs while keeping a low weight.^4−7^ Many siliceous skeleton species, such as diatoms and sea sponges, also show porous lightweight structures and remarkably high strength, which arise—as for other biological materials—from the hierarchical arrangement of different structural features at their relevant length scales.^8−10^

The need for lightweight, functional structures and the rapid development of additive manufacturing (AM) has boosted the research of architected cellular materials, leading to periodic microlattices with graded porosity and truss structures optimized for specific loadings. Thus, by combining optimized cellular architectures with high-performance constituent materials (e.g., metals, ceramics), several high-strength/high-stiffness lightweight materials can be fabricated. Such materials find applications in different fields, because of a wide range of structure- and material-driven properties (e.g., acoustic, thermal, and biological).^11,12^ Besides the most common existing AM technologies, which are limited to ∼20–50 μm resolution, recent lithography-based processes also offer an efficient route to manufacture complex microarchitected and nanoarchitected metals with a submicrometer resolution (∼100 nm).^13^ For example, multistep nanofabrication processes involving a combination of two-photon lithography, direct laser writing, and atomic layer deposition allow the fabrication of hollow ceramic nanolattices that mimic the length scales and hierarchy of biological materials.^14−16^ Architected materials have proved to be a very effective way of making materials characterized by extremely high stiffness-to-weight ratio or unusual Poisson’s ratio. Much of the work in this area has focused on tailoring properties like stiffness and density.^17^ Yet much remains unknown about the postyield properties (e.g., critical load to failure). Injeti et al.^18^ suggested a method to optimize the specific load to failure independently of specific stiffness and density by adding local internal prestress in selected regions of truss-like structures. A similar approach could also be used to control locations and failure paths in architected materials.

Recent studies have mostly focused on the design, fabrication, and modeling of perfect lattice materials. However, in some practical applications, lattice structures contain local stress-raisers (e.g., holes, notches, inclusions), often arising from the manufacturing process.^19−21^ Such defects can significantly knock-down the macroscopic ductility and strength of these lattices. Similarly, in additively manufactured lattices, intrinsic defects can alter the stress and strain distribution, leading to premature failure. Accounting for these manufacturing-induced defects is paramount for a proper and more realistic design phase. Recently, Li et al.^22^ proposed a local reinforcement technique, based on a spatially nonuniform waviness distribution of struts in the vicinity of the notch, to reduce the notch sensitivity and improve the macroscopic strength and ductility of the lattice. A characteristic feature of additively manufactured lattices is the material concentration in the nodal regions, which generally results in an increased stiffness of those areas with respect to the struts.^21^ To account for this effect in beam-based finite element models, a commonly employed approach is to increase the thickness of the beam element in the neighborhood of the nodal regions.^23,24^ As an example, Labeas and Sunaric^23^ increased the strut diameter by 40% in the nodal areas, whereas Smith et al.^24^ did so by 30%. Alternatively, the same effect can be obtained by acting on the element material, as done by Luxner et al.,^25^ who increased the Young’s modulus of the beam elements within a spherical domain around the node area. The radius of the domain was taken equal to the strut radius. The actual manufacturing irregularities are often considered as a local thickness variation of the strut. From the modeling point of view, this can be obtained by defining elements with different diameters.^25−27^ For instance, Campoli et al.^25^ used beam models to study the mechanical behavior of open-cell porous biomaterials and assumed a variable strut thickness, according to a Gaussian distribution, with the mean value corresponding to the nominal diameter of the strut and the standard deviation determined from scanning electron microscopy (SEM) analyses. The same approach was followed by Zargarian et al.,^27^ whose study was focused on the fatigue behavior of titanium scaffolds fabricated by selective laser melting (SLM). Karamooz Ravari et al.^26^ also considered strut elements with a variable thickness (defined through statistical analysis on the measurements taken on the actual cells) to describe the effect of manufacturing irregularities of lattice structures fabricated by fused deposition modeling (FDM). Yet, they used a similar approach on both beam and solid numerical models. Similarly, we propose a numerical framework accounting for AM-induced defects based on the defects experimentally observed in our samples. Our modeling approach is based on experimental evidence and is supported by similar approaches in the literature.^23−25^ However, it is an alternative and novel procedure, validated on our experimental data. Moreover, it is simple, as it does not require a partition to assign different moduli or thicknesses along each strut. Thus, it could be easily implemented in the design of large arrays, also providing the basis for future coarse-grained models.

The effective properties of low-density lattice materials are also defined by their topology or cellular architecture (i.e., the spatial configuration of voids and solid) and by the mechanical properties of the solid constituent (e.g., stiffness, strength, etc.). Ultralow-density structures, such as aerogels and polymeric foams, present a stochastic cellular architecture, which confers high specific surface area but limited specific mechanical properties if compared with those of the bulk constituents.^28^ A large-scale classic example, the Eiffel Tower, shows that introducing a hierarchical framework, which is a hallmark of natural structures, can significantly improve the material deployment making the construction structurally efficient.^29^ Indeed, the Eiffel Tower has a relative density (i.e., the density of the structure divided by the density of the material it is made of) just 1.2 × 10^–3^ times that of iron, which is weaker than structural steel. Yet, introducing hierarchy often encounters manufacturing-induced limitations, despite the recent advances in manufacturing techniques. For this reason, most of the bioinspired designs generally focus on a specific level of hierarchy^30−35^ or a few hierarchical levels.^36^ Pham et al.^37^ recently showed a way to overcome this limitation by designing mesoscale metal lattice structures that mimic crystallographic microstructures. The proposed approach will allow one to implement the hardening mechanisms found in crystalline materials at multiple length scales, i.e., the constituent material level and the architecture level, yielding to highly damage-tolerant materials and offering new ways of studying complex metallurgical phenomena. Similarly, our long-term goal is to implement the characteristic mechanisms occurring in single-crystal lattices into 3D-printed polymer cells and combine them with architecture-driven mechanisms and failure mechanisms of polymers. When crystals with different orientations meet, they create grain boundaries at the interface. In nanocrystalline solids, grain boundaries become a significant volume fraction of the material, with profound effects on properties such as diffusion and plasticity. Thus, in large arrays, plasticity and hardening can be driven by lattice-inspired unit cells, and the combination of different unit cell types can be used for fine-tuning the global mechanical behavior. The use of polymer printing will also enable implementing the typical mechanisms of metallic materials into polymeric architectures and benefiting from diverse mechanisms. The mechanical behavior of such a new class of architected materials that mimic crystalline microstructure at mesoscale (meta-crystals) will be governed at different levels: a material level (the crystalline microstructure), a geometrical level (architected crystal-like mesostructures), and a manufacturing level (i.e., the quality of lattice struts). Yet, as the authors recently showed,^38^ significant processing defects in the printed meta-crystals can strongly reduce the properties and influence the overall behavior. Thus, the design of such crystal-inspired structure should account for manufacturing-induced defects, which may depend on the base materials (e.g., metals vs polymers) and on the manufacturing techniques.

In this work, drawing inspiration from the cubic Bravais lattices,^39^ we design three different unit cells, where the struts aim to mimic the bonds and the nodes aim to mimic the atoms positions. We use a comprehensive approach, including 3D printing, experimental testing, and numerical simulations to assess the behavior of each unit cell, also probing the effect of printing parameters, such as the layer thickness and the printing direction. To provide useful and accurate models for the design of large arrays, we suggest a method that accounts for manufacturing-induced defects. The proposed method could be implemented in both 3D solid and beam models, providing a good comparison with experimental data. The results demonstrate how the printing-induced defects influence the overall mechanical behavior of the cells and the importance of accounting for their effect in the modeling framework.

## 2 Materials and Methods

The study involves four steps:

- *Sample design*, including both the specimens for materials characterization and the Bravais lattice-inspired unit cells.
- *Sample manufacturing*, which involves the 3D printing of tensile samples, compressive samples, and unit cells. Here, different printing parameters, such as the printing direction and the layer thickness, are investigated.
- *Sample testing*, which involves the experimental mechanical characterization of both the bulk material and the unit cells.
- *Numerical simulations* of both the unit cells and the cell-based arrays, based on two modeling approaches.

**Figure 1:**
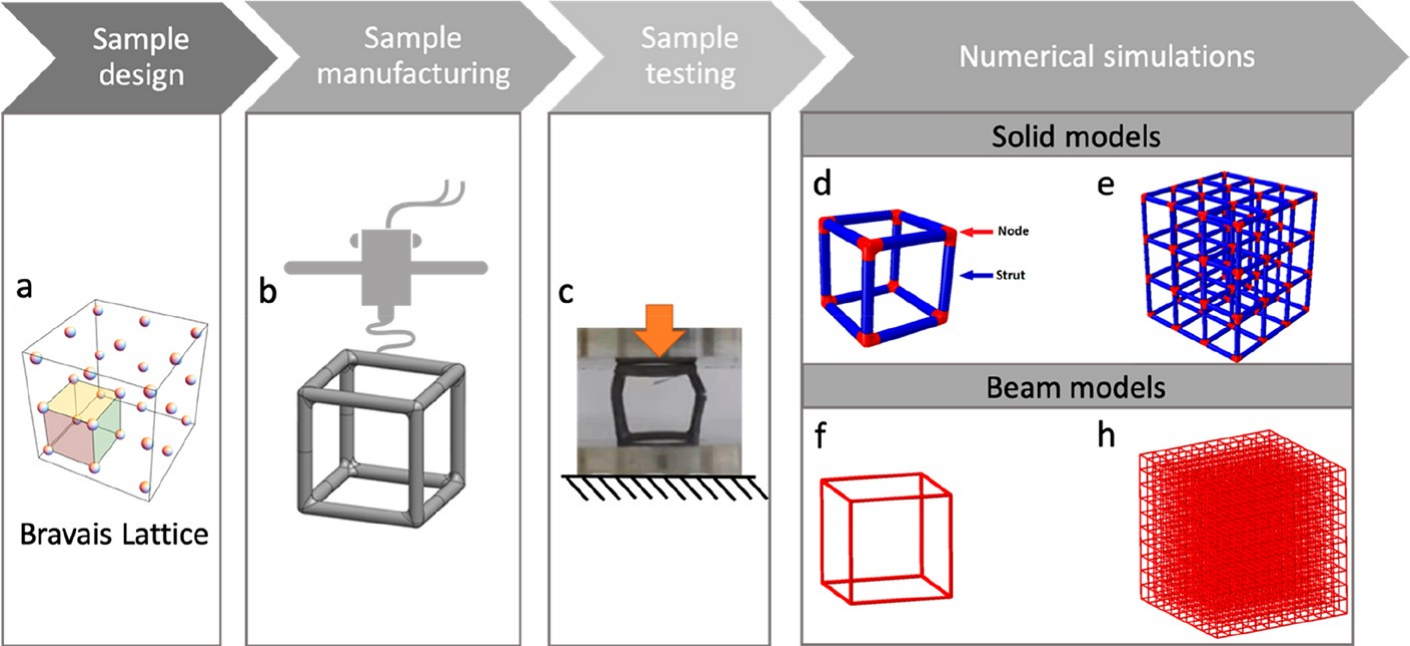
Framework of the study: (a) inspiration from the Bravais cubic lattice; (b) cell design and 3D printing; (c) mechanical testing; (d,e) 3D solid FE models of (d) unit cell and (e) array; (f, h) 3D beam FE models of the (f) unit cell and (h) array. Image (a) generated by the authors using Wolfram Demonstration Project, © 2021 Wolfram.

The approach followed in this study is represented as a schematic in Figure 1, where we have highlighted these four phases (i.e., sample design, sample manufacturing, sample testing, and numerical simulations).

### 2.1 Sample Design

All the samples are modeled using *SOLIDWORKS2019* (Dassault Systems), exported as “.stl” files, and then converted into “.gcode” files through the software Ultimaker *Cura*.

#### Tensile Specimens

Dogbone tensile specimens (4 mm thick) are designed according to the standards ASTM D638-14.^40^ Two sample geometries are designed. Size and geometries are provided in the Supporting Information.

#### Compression Specimens

Cylindrical compression specimens with a diameter equal to 12.7 mm and a height of 25.4 mm are designed according to the standard ASTM D695-15.^41^

#### Cells

The geometries of the cells are inspired by the following cubic Bravais lattices: SC (simple cubic), BCC (body-centered cubic), and FCC (face-centered cubic). The struts represent the atomic bonds, whereas the intersection points, here called nodes, represent the position of the atoms in each Bravais lattice (Figure 1a, d). The cells have a nominal size *l* = 16.00 mm, with a strut diameter *d* = 1.75 mm and a nominal volume *V* = 4096.00 mm^3^. To avoid local stress concentration during the compression, we added a fillet with a radius of 0.70 mm at the intersection of two struts. The cell’s size is chosen based on preliminary studies on the printing resolution and the machines available in our lab for mechanical testing.

### 2.2 Sample Manufacturing and Printing Parameters

All the samples are manufactured by means of an Ultimaker 3 3D printer, which uses the FFF (fused filament fabrication) technology. The printer has a dual-extrusion printing head, a maximum building volume equal to 197 × 215 × 200 mm, and a layer resolution of 200–20 μm corresponding to a nozzle with a diameter equal to 0.4 mm. The material used for all the samples is the Ultimaker Silver Metallic PLA (RAL 9006).^42^ In this study, we fixed the following parameters: nozzle diameter (0.4 mm), infill (100%), curing temperature (room temperature), and we studied the effect of layer resolution (i.e., layer height) and printing orientation. Considering the FFF-induced anisotropy, we wanted to probe the effect of the printing direction. Also, we wanted to experimentally explore whether these parameters influence the sample mechanical properties and we wanted to define a specific set of parameters to be used for the current and future studies.

#### Tensile Specimens

The tensile specimens are printed considering the following parameters: (i) Three orientations (*X*–*Y*–*Z*), where *X* and *Y* are the in-plane directions, longitudinally and orthogonally to the main specimen axis, respectively, and Z is the out-of-plane direction, (ii) Two different layer heights (0.15 mm and 0.20 mm), and (iii) An infill density of 100%. In this study, four different sample families are considered: X015, X02, Y02, and Z02. The letter indicates the printing direction, whereas the number indicates the layer height. For each family, three samples are printed.

#### Compression Specimens

Compression specimens are printed considering two-layer heights and an infill density of 100%. For this study, we consider three cylindrical samples with layer height 0.15 mm, named CI015, and three cylindrical samples with layer height 0.20 mm, named CI02.

#### Cells

**Figure 2:**
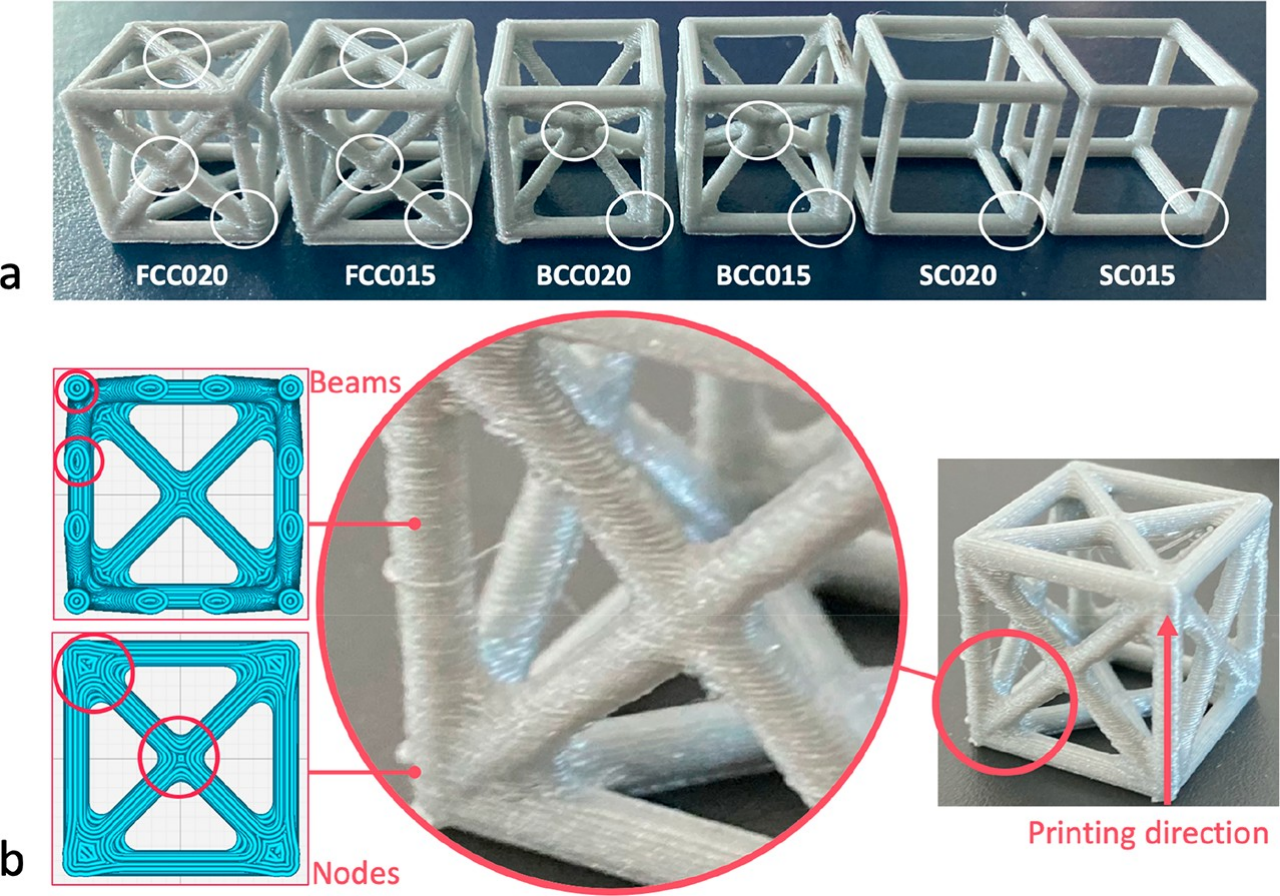
(a) Six families of 3D-printed unit cells analyzed in this study (FCC02, FCC015, BCC02, BCC015, SC02, SC015). The letters indicate the lattice type (SC, simple cubic, BCC, body-centered cubic, and FCC, face-centered cubic), whereas the number indicates the layer height (015 = 0.15 mm and 02 = 0.20 mm). In this picture, the different types of nodes that characterize each cell are highlighted through a circle. (b) Magnification on the printing quality showing higher deposition of material at the nodes with respect to the struts and more visible defects in the struts. On the left, sliced view of the FCC sample at two different heights: whereas the printing path related to each vertical or inclined beam is mainly represented by circular or elliptic paths, at the node, the material is less anisotropic because of the more complex printing path.

Cells are printed using two different layer heights (0.15 mm and 0.20 mm) and as 100% solid samples (considering their small dimensions, the samples are generated mainly as inner and outer walls). We consider six distinct cell families, distinguished by the geometry and the layer height: SC015, SC02, BCC015, BCC02, FCC015, and FCC02. For the nomenclature, we consider the cell acronym, followed by the layer height. Each family includes ten samples. These six cell families are shown in Figure 2a. They are printed using the following parameters: printing speed (80 and 70 mm/s for 0.15 and 0.20 mm layer height, respectively), printing temperature (200 and 205 °C for 0.15 and 0.20 mm layer height, respectively), and build plate temperature (70 °C for the FCC and BCC, 60 °C for the SC).

### 2.3 Mechanical Testing

To characterize the PLA, we carried out tensile and compressive tests on the bulk material. To investigate the behavior of each unit cell, we carried out compressive tests on each cell. Although there is no standard procedure suggested for the compressive testing of the cells, the same experimental setup is adopted to ensure a proper comparison among all the families.

#### Tensile Specimens

Displacement-controlled tensile tests are carried out at room temperature using an MTS Alliance RT/100 universal tensile testing machine with a 100 kN load cell in place. A crosshead speed of 1 mm/min, corresponding to a strain rate of 3 × 10^–3^ s^–1^, is adopted until failure. The displacement is measured through an extensometer MTS 635.25F-05 with a gauge length of 25 mm.

#### Compression Specimens

Displacement-controlled compression tests are carried out at room temperature using an MTS RF/150 universal tensile testing machine with a 150 kN load cell in place. A crosshead speed of 1.3 mm/min, corresponding to a strain rate of 7 × 10^–3^ s^–1^, is employed until failure, and an STM 632.26F-2X deflectometer with a gauge length of 8 mm is used to evaluate the displacement.

#### Cells

Displacement-controlled compression tests are carried out at room temperature on all the cells using a crosshead speed of 1 mm/min, corresponding to a strain rate of 2 × 10^–3^ s^–1^. To evaluate the effect of the printing vs loading direction, each family is divided into two groups:

- L, samples tested longitudinally with respect to the printing direction;
- T, samples tested transversally with respect to the printing direction.

**Figure 3:**
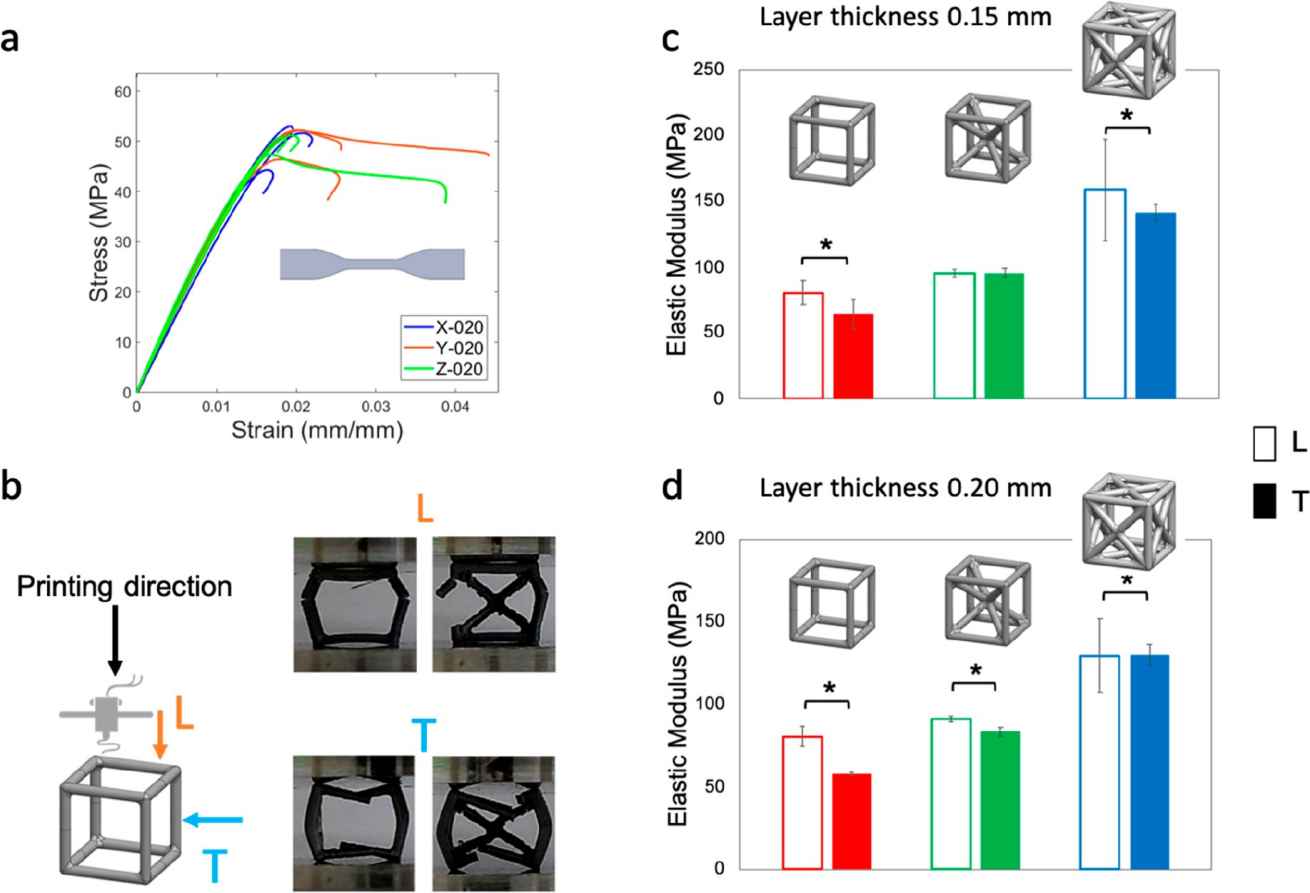
Effect of the loading vs printing direction on (a) dogbone tensile samples, (b) failure mode (SC vs BCC), (c) cells printed with 0.15 mm thickness layer, and (d) cells printed with 0.20 mm thickness layer. Empty and filled bar indicate L (longitudinal) and T (transversal) loading directions, respectively, vs the printing one. *(*p*-value <0.05).

The printing/loading directions are indicated in Figure 3b. Each group includes five repetitions. Because of the complex geometry of the cells and the difficulty in placing an extensometer, the displacement is measured through the crosshead and a gauge length, *l*_0_ = 16 mm, is used to calculate the strain. Compression tests of SC and BCC cells are carried out on an MTS Synergie 200 electromechanical machine with a 1 kN load cell in place and MTS 643 compression plates to ensure pure axial loading. Compression tests of FCC are carried out on an MTS RF/150 universal tensile testing machine with a 150 kN load cell in place.

For all the cells, the nominal stress, σ, is calculated as follows: , where *F* is the force measured by the load cell and *A*_0_ the nominal (or apparent) area. The nominal strain, ε, is calculated as , where *l*_0_ is the original length of the specimen, and *l′* is the final length. The stiffness, *K*, is calculated as . Yielding stress and strain cannot be easily estimated for these cells. Hence, the yielding point is defined by the intercept between the stress–strain curve and a straight line having the same slope of that identifying the Elastic modulus and, as origin, ε = 0.2%. As an indication of toughness, the area underneath the stress–strain curve, representing the strain energy per unit of volume, , is calculated. Failure is defined by a 50% load drop.

#### Statistical Analyses

Statistical analysis is carried out, and a *p*-value <0.05 is assumed as the significant level. ANOVA is carried out to determine whether there is any statistically significant difference between the means of the different groups. Multiple comparison tests for all pairwise differences between the groups are carried out by the Student’s *t* test (*p*-value <0.05).

### 2.4 Numerical Simulations

Finite Element (FE) simulations are performed to study the mechanical behavior of each unit cell and to predict the performance of cell-based arrays. All the simulations are carried out using Abaqus CAE 6.14 (Dassault Systems). For the cells, we consider two types of models, the solid model and the beam one. All the analyses carried out are linear elastic.

#### Cells: Solid Models

3D solid models are created for both the single unit cells and the cell-based arrays to study how each topology affects the stress distribution and the overall mechanical behavior. The model geometries are created with *SOLIDWORKS 2019*, then imported as “.step” file into Abaqus. Appropriate partitions are used to separate the nodes from the struts, ensuring a better discretization and a more regular mesh. As material properties, we initially assign a Young’s modulus equal to 2346.5 MPa and a Poisson ratio ν = 0.36, both taken from the datasheet of the PLA material.^42^ We carry out a preliminary mesh convergence study, allowing us to choose the appropriate mesh size and estimate the mechanical behavior of the cells. Preliminary numerical simulations are carried out before testing, thus using the properties of the material of the datasheet. After experimental testing, new simulations are carried out using the experimentally determined elastic modulus. Ten-node tetrahedral elements with reduced integration (C3D10R) and an average global size of 0.5 mm are adopted. The mesh convergence study is carried out by considering four different element type combinations (for nodes and struts) and by systematically varying the average element size (from 0.6 to 0.2 mm). To mimic the experimental compression tests, we add two discrete rigid plates with contact interaction between the plates and the surfaces of the cells. A general contact with penalty formulation is adopted (friction coefficient equal to 0.2 for the tangential behavior and hard contact for the normal behavior). Boundary conditions are applied through two reference points: an encaster is applied to the bottom plate and a vertical displacement (0.5 mm) to the top plate. The strain, ε, is calculated considering the variation of the distance between the two reference points, divided by the nominal side length, *l*_0_ = 16 mm. The nominal stress is calculated by dividing the reaction force at the encaster by the cross-section (*A*_0_ = 256 mm^2^). The outcome of preliminary simulations shows that the models are much stiffer than the experimental results. By observing the printing procedure and the printing quality of the cell-like samples, we notice a different printing quality between the struts and nodes, which is likely to influence the local and global mechanical properties. To accurately represent this phenomenon and obtain a model comparable with the experimental data, we assign different elastic moduli to nodes and struts, corresponding to different percentages (ranging from 100% to 50%) of the experimentally determined elastic modulus (3385 MPa). The calibration procedure involves several simulations. We systematically vary the elastic properties assigned to the struts and the nodes to find the combination that best approximates the experimental results. More details are provided as Supporting Information. The calibrated model of the unit cells is then used to build 2 × 2 × 2 and 3 × 3 × 3 arrays for each cell type. To reduce the computational effort, for the BCC-3 × 3 × 3 and FCC-3 × 3 × 3 models, we used four-node tetrahedral elements with full integration (C3D4), instead of the C3D10R.

#### Cells: Beam Models

3D beam models are created for both the single unit cells and the cell-based arrays. The struts are modeled as beams, 14.25 mm long, having a circular profile with a radius of 1.75 mm. For the mesh, three-node quadratic beam elements (B32) with seed size 0.5 mm are chosen. To reproduce the compression test, we apply a distributed vertical load to all the edge wires, while the four edges of the opposite face are constrained in the direction of the applied load. Being the beam model a simplified representation, which neglects the node geometry and the effect of the fillet, only the material of the struts is considered. By following the same approach adopted for the solid model, for the beam model calibration, different elastic moduli, corresponding to a ratio of the experimentally determined elastic modulus (in the range of 100 to 50%), are assigned to the struts. Given the lower computational cost of these simulations, cell-based array models of up to 20 cells per side are analyzed. In detail, arrays having 2, 3, 5, 7, 10, 15, and 20 cells per side are analyzed. Moreover, to study the effect of density on the overall mechanical behavior of large arrays, additional 10 × 10 × 10 models are created considering the strut size reduced by 30, 40, and 50%, thus adopting a radius of 1.31, 1.05, and 0.875 mm.

#### Data Postprocessing

We consider: *l*_0_ = the initial length of each sample; *A*_0_ = the nominal or apparent cross-section; *m*, the mass of the cellular/lattice material; *m*_s_, the mass of a solid made of the same constituent material; *V*, the nominal or apparent volume; ρ = *m*/*V*, the apparent density of the cellular/lattice material; *ρ*_s_ = *m*_s_/*V*, the density of the constituent solid; , relative density defined as the ratio of the cellular material density to the density of a solid made of the same constituent material.  relative elastic modulus defined as the ratio of the elastic modulus of the cellular material to the elastic modulus of a solid made of the same constituent material.

## 3 Results and Discussion

The experimentally determined Young’s modulus, calculated considering all the dogbone samples with different orientations and different layer thicknesses, is 3385 ± 180 MPa, whereas the compressive elastic modulus, calculated considering all the cylinders with different layer thicknesses, is 3028 ± 281 MPa. For the unit cells, we determined the following elastic moduli (considering all the samples with different layer thicknesses): 71.05 ± 8.23 MPa for SC cells, 91.43 ± 5.19 MPa for BCC cells, and 139.8 ± 18.4 MPa for FCC cells. The other mechanical properties and the stress–strain curves of all the cells are included as Supporting Information.

### 3.1 Effect of Printing Direction

The experimental tensile tests carried out on the bulk material show no effect of the printing vs loading direction, except for the strain at the breakage, which is higher for samples printed in the *Y*- and *Z*-directions (Figure 3a). This is confirmed by the ANOVA analysis carried out considering the three independent sample groups with different printing orientations (X02, Y02, Z02) and by the Student’s *t* tests performed on a series of pair comparisons on independent sample groups (X02-Y02, Y02-Z02, X02-Z02). All the dogbone samples show the same failure, orthogonally to the load applications and at the end of the nominal section. On the cells, instead, we notice a clear effect of the printing direction, both on the failure mode and on the elastic modulus: cells loaded longitudinally to the printing direction fail by buckling of the vertical struts, whereas cells loaded orthogonally to the printing direction fail by bending of the horizontal struts (Figure 3b). Also, the cells loaded longitudinally to the printing direction show a significantly higher elastic modulus (*p*-value <0.05) compared to the cells loaded orthogonally to the printing direction (Figure 3c, d).

### 3.2 Layer Thickness Effect

**Figure 4:**
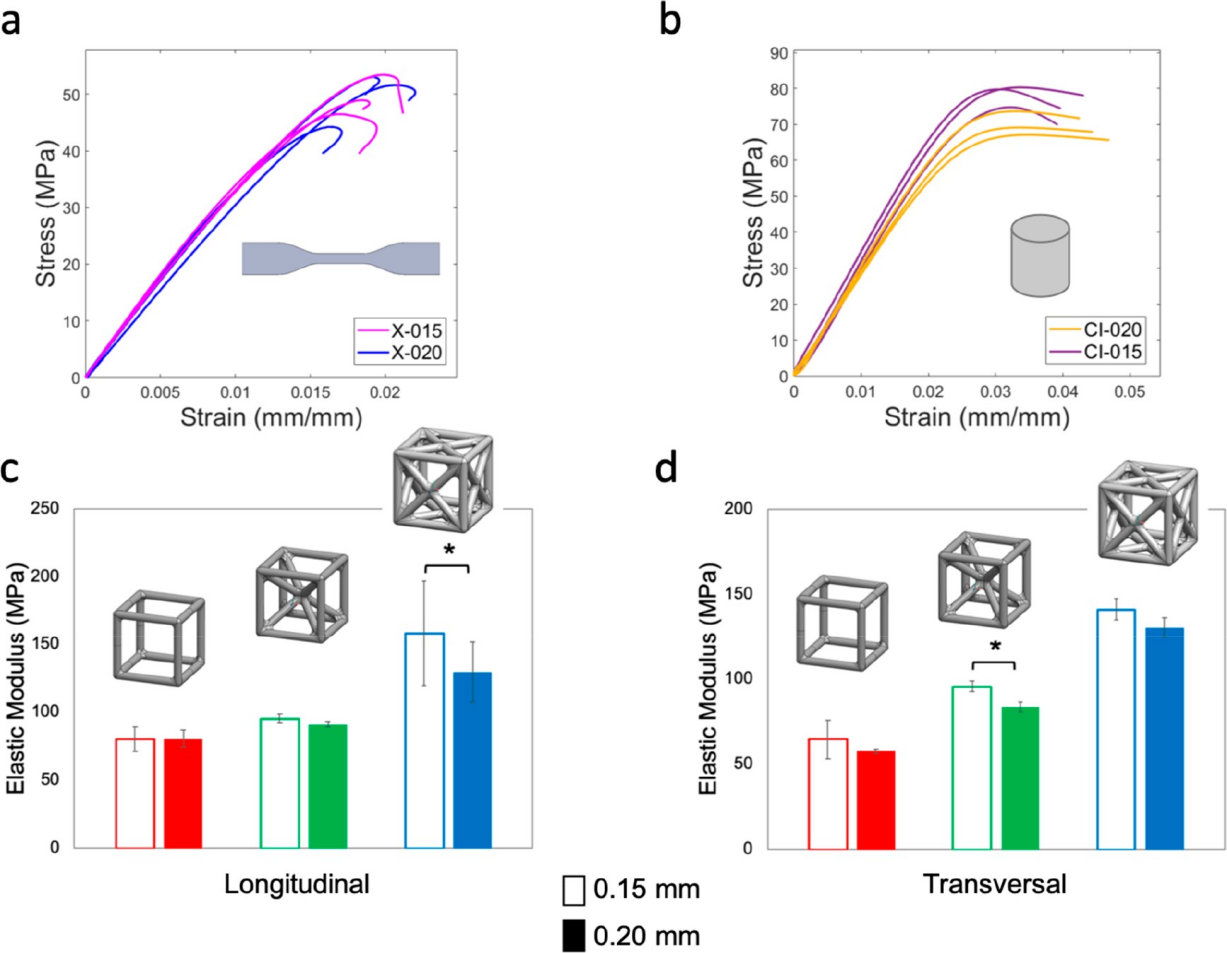
Layer thickness effect under (a) tensile and (b) compressive loading for the bulk PLA. Layer thickness effect on: (c) cells tested longitudinally and (d) transversally to the printing direction. Empty and filled bars indicate 0.15 mm and 0.20 mm layer thickness, respectively. *(*p*-value <0.05).

The outcome of the mechanical tensile and compressive testing shows no clear effect of the layer thickness (Figure 4), neither for the bulk materials nor for the cells. This is confirmed by the Student’s *t* tests performed on a series of pair comparisons on independent sample groups with layer thickness 0.15 mm and 0.20 mm. Only for two cases, FCC-L and BCC-T, we notice a significant difference, with higher modulus for cells printed with 0.15 mm layer thickness vs cells printed with 0.20 mm layer thickness. The failure mode is also not affected by the layer thickness. However, we notice by bare eye that a higher resolution (0.15 mm) generally presents more visible defects (e.g., yarns unraveling at fillets).

### 3.3 Numerical Simulations

**Figure 5:**
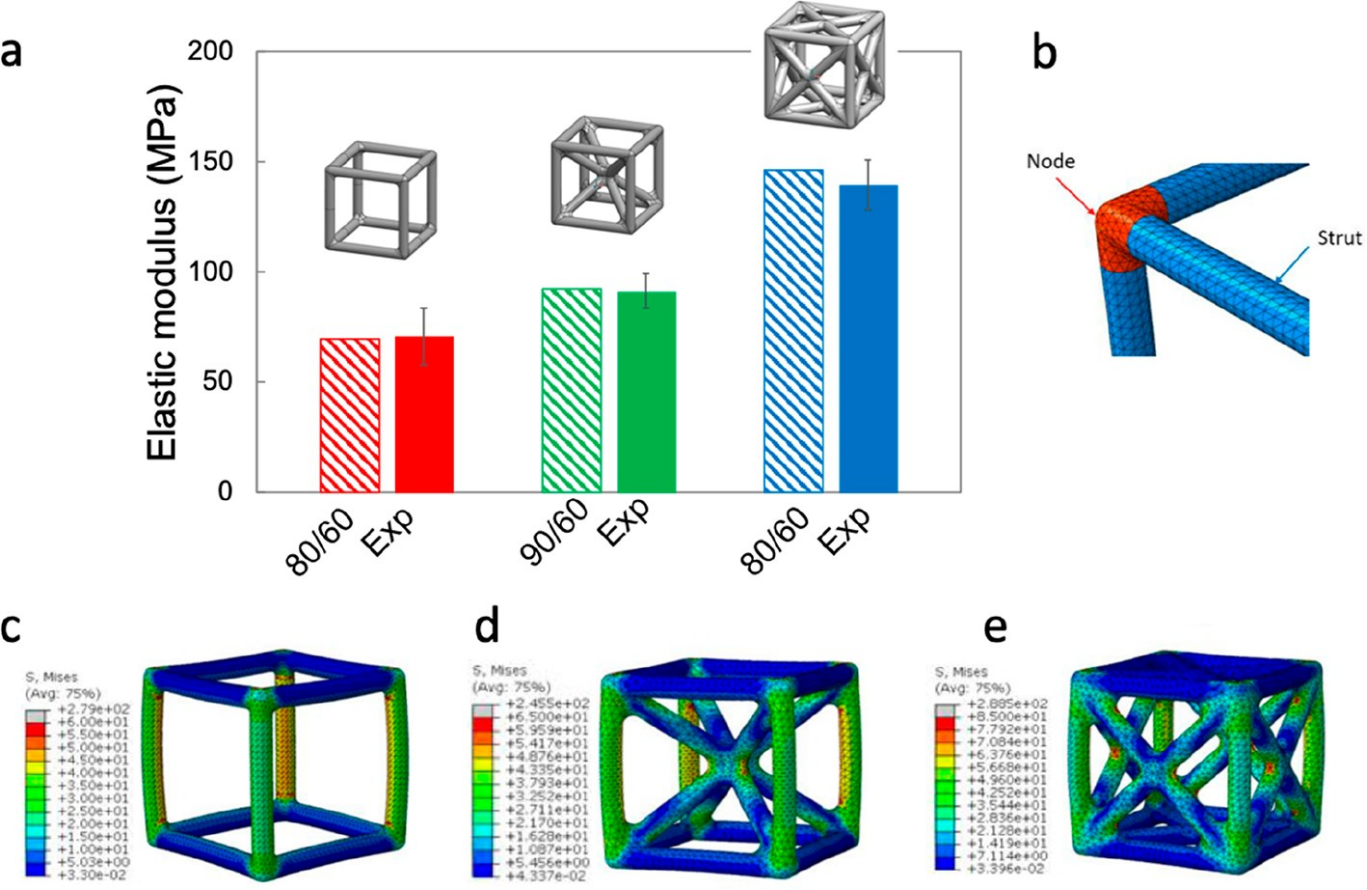
(a) Comparison between experimental and numerical (3D solid models) data in terms of elastic moduli. (b) Partition of nodes and struts. Von Mises stress distribution on (c) SC (single cubic), (d) BCC (body-centered cubic), and (e) FCC (face-centered cubic) solid FE models.

By observing the samples by bare eye, we notice a lower printing quality in cells (Figure 2) with respect to cylinder and dogbone samples (Figure S1b). Also, a different printing quality can be noticed within each cell (Figure 2). In particular, the struts are more likely to be affected by defects with respect to the nodes (e.g., interface defects in-between the deposited layers that can lead to layer–layer debonding). We observe a higher deposition of material at the nodes with respect to the struts and more visible defects in the struts (Figure 2b). Moreover, the struts present a higher anisotropy, because of the more complex printing path. Figure 2b shows a sliced view of the FCC sample at two different heights: although the printing path related to each vertical or inclined beam is mainly represented by circular or elliptic paths, at the node, the material is less anisotropic because of the more complex printing path. To account for this effect, we assigned different elastic moduli to nodes and struts (Figure 5b). We found that the combination that best approximates the elastic behavior of the unit cell is 80–60 (80% of the experimental elastic modulus for the nodes and 60% for the struts) for SC and FCC and 90–80 for BCC (Figure 5a), with a difference between the numerical and the experimental elastic moduli of 1.3, 1.2, and 4.7%, for SC, BCC, and FCC, respectively. For all the cells, we notice that the vertical struts are more stressed with higher stress concentration at the fillets (Figure 5c–e).

**Figure 6:**
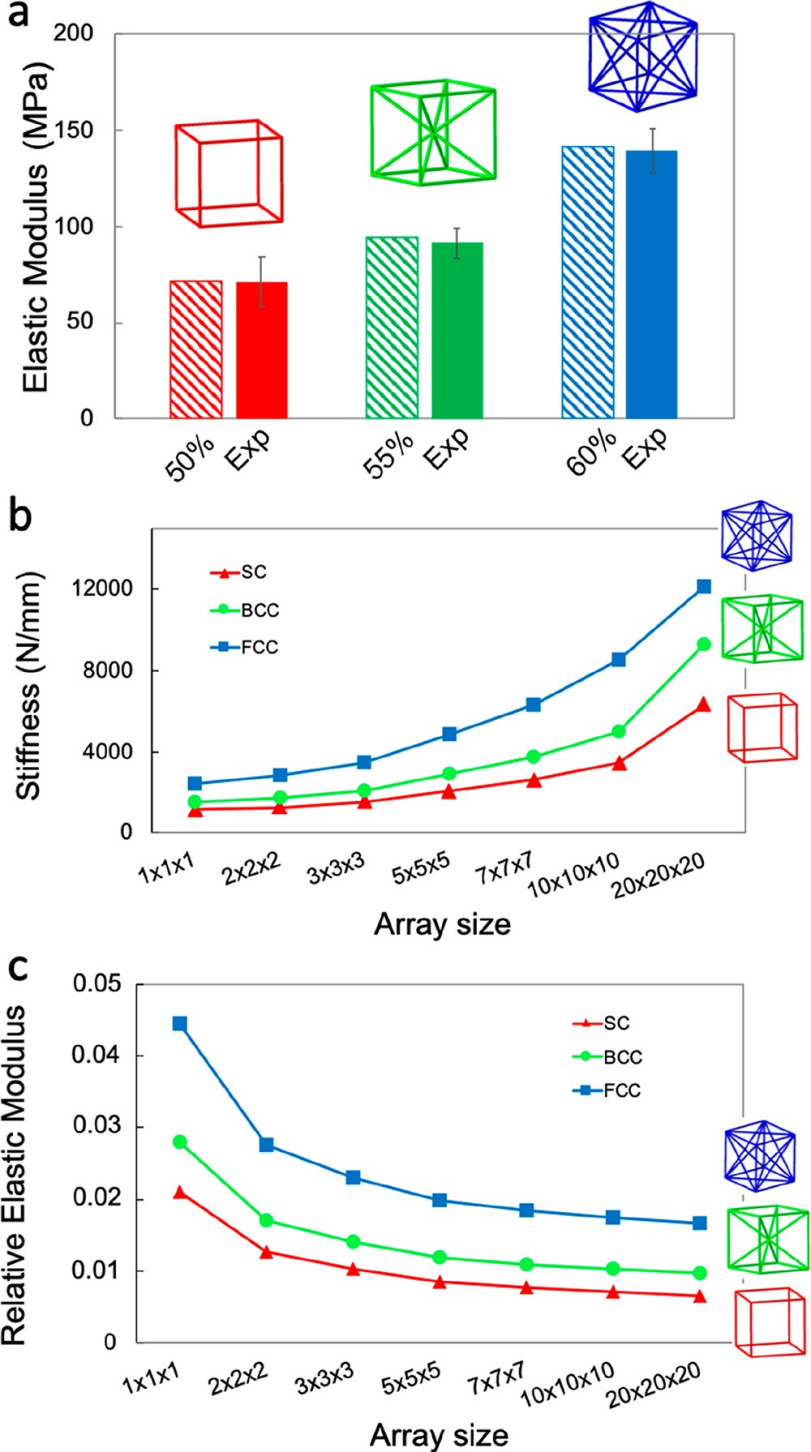
(a) Comparison between experimental and numerical (3D beam models) data in terms of elastic moduli. (b) Stiffness trend for different arrays showing an exponential increase in the stiffness. (c) Relative elastic modulus trend for different arrays showing a plateau reached for an array size larger than 7 × 7 × 7.

In the beam model, which is a simplified FE model, where the geometry of the node and the fillet are neglected, we only consider the struts, assigning a reduced elastic modulus (i.e., a ratio of the experimental elastic modulus). We find that the ratios that best approximate the elastic behavior of the unit cell are 50, 55, and 60% for SC, FCC, and BCC, respectively (Figure 6a). The differences between the numerical and the experimental elastic moduli are 0.6, 3.4, and 1.47% for SC, BCC, and FCC, respectively. By increasing the array size, we notice how the stiffness increases exponentially (Figure 6b), whereas the relative elastic moduli tend to reach a plateau above a 7 × 7 × 7 array size (Figure 6c). The latter means that arrays larger than 7 × 7 × 7 can provide a good approximation of periodic lattice-like structures. The elastic modulus of a single or reduced number of cells is influenced by the edge effect. By increasing the number of cells, we approach the condition of a periodic lattice, here described by the plateau of the elastic moduli. Considering that each strut can be shared by up to four adjacent cells, as the number of cells increases, the number of struts that arise from the intersection of four adjacent cells increases and tends to best approximate the whole volume lattices, making the boundary effect negligible. This is also confirmed by many studies^43−46^ where arrays with size larger than 5 × 5 × 5 are generally used. The relative modulus is calculated by dividing the numerical modulus of each array by the Young’s modulus of the bulk material that was used as input data for the numerical models (the reduced modulus assigned to the struts, i.e., 50, 55, and 60% of the experimentally measured Young’s modulus, for SC, FCC, and BCC, respectively).

**Figure 7:**
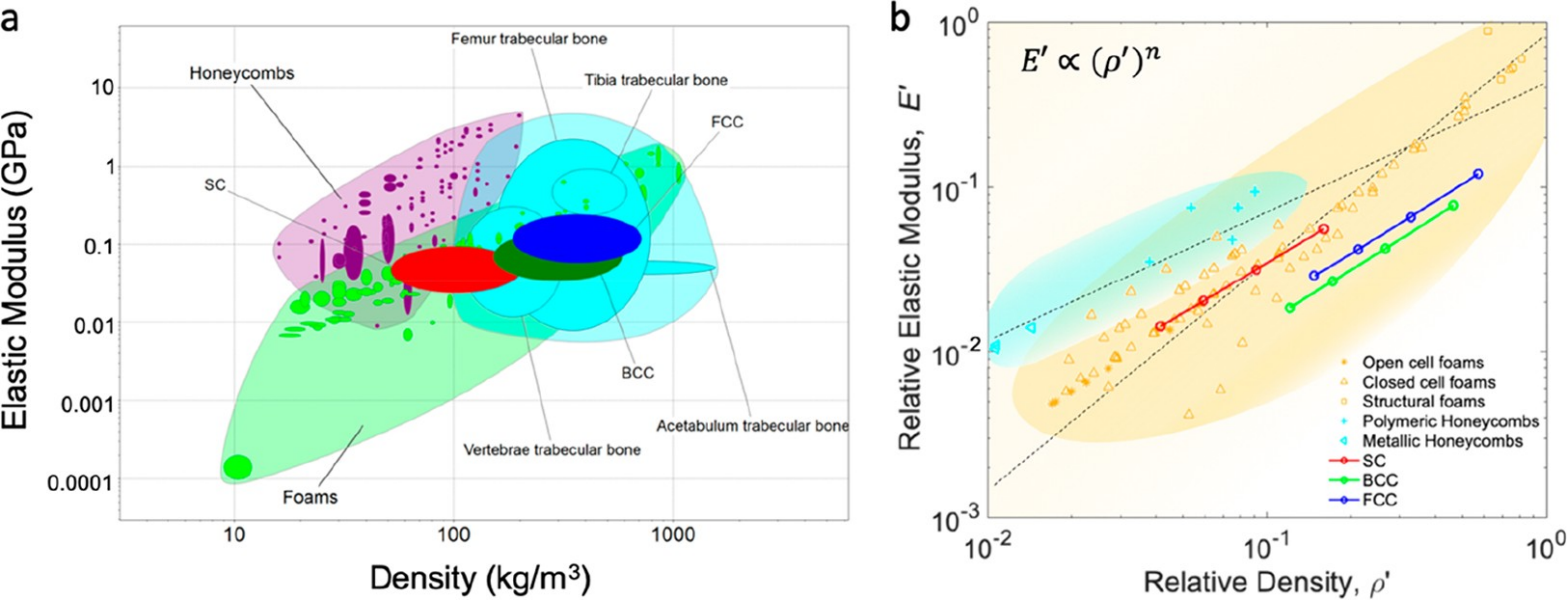
(a) Compressive modulus vs density for different lightweight materials families and comparison with the studied lattices (SC, BCC, and FCC). (b) Relative compressive elastic modulus (i.e., the measured compressive elastic modulus of the lattice divided by the compressive elastic modulus of the constituent solid) of the studied 10 × 10 × 10 lattices as a function of the relative density and comparison with foams and honeycombs. The two trendlines approximate the different behavior of honeycomb and foams. Data related to the lattices studied in this paper refer to numerical simulations. Data for foams, honeycombs, and bone are taken from the CES Edu Pack database.

The effect of the relative density on the overall mechanical behavior of large arrays is shown in Figure 7. By plotting the relative compressive modulus, *E′* of various 10 × 10 × 10 beam-like lattice models versus their relative density, ρ*′*, we show that the modulus scales with (ρ/ρ_s_)^*n*^ (Figure 7b) where *n* is approximately 1. For the calculations, we kept the array size constant (10 × 10 × 10) and increased the strut size by 20, 50, and 100%.
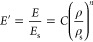
1In particular, we find
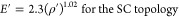
2
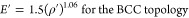
3
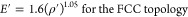
4The scaling laws for each cell topology, indicated in Figure 7b, show a stretch-dominated behavior similar to that of honeycomb-like structures tested in out-of-plane direction (*n* = 0.81 and *n* = 0.93 for polymeric and metallic honeycomb respectively), which are considered materials with high structural efficiency. Bending-dominated structures, such as structural foams instead, scale with ∼(ρ/ρ_s_)^1.74^.

The linear scaling of the relative modulus of the studied periodic lattices is also found in octet-truss lattices,^47−49^ but not in microlattices (with an exponent *n* = 2), where the ultrathin-shell hollow struts promote localized bending deformation.^16^ Contrarily, very ultralow-density materials (characterized by densities <10 mg/cm^3)^, such as aerogels and carbon nanotube (CNT) foams, reveal a steeper scaling of (*E*/*E*_s_) ∼ (ρ/ρ_s_)^3^ due to inefficient load transfer between ligaments.^50,51^ The case of trabecular bone, instead, is somehow particular, as the scaling law depends on the anatomical site. The exponent, *n*, for the human bone falls in the range of 1.49–2.18,^52^ which might suggest that deformation mechanisms are similar across sites and involve appreciable bending. Nevertheless, the limited density range shown by each site alone makes the differences between predicted values from linear and power law models negligible.

In this study, the results provided for large arrays are theoretical. Considering the outcome of experimental testing and the effect of printing direction on the cell failure mode, we believe that the lattice tested in the longitudinal direction will fail in a stretching-dominated mode. In contrast, those that are tested in transversal direction will also show bending-dominated deformation. From a numerical point of view, a more sophisticated model accounting for the different stiffnesses of the beams with respect to the printing orientation could be implemented. On another note, these results can provide the basis for implementing a homogenization-based approach. Indeed, a deeper understanding of the structure–property relationship of each cell family can be used to design voxel-based homogenized models.

## 4 Conclusion

Inspired by Nature and harnessing 3D printing, mechanical testing, and numerical modeling, we study the structure–property relationship of periodic lattices inspired by the cubic Bravais crystals. In particular, the simple cubic (SC), body-centered cubic (BCC), and face-centered cubic (FCC) lattices are studied. Finite element models based on both 3D solid and beam elements are developed to describe the structural response of the unit cells and the cell arrays. Mechanical testing of 3D-printed structures is used to investigate the influence of different printing parameters and embed their effects in the numerical simulation framework.

We notice no clear effect of the layer thickness on the mechanical properties of bulk material and lattice structures. The printing direction effect, although negligible in solid samples, becomes relevant when printing lattice structures. Indeed, the local mechanical properties of the unit cells are largely affected by the printing-induced defects, leading to different stiffnesses of struts and nodes. This phenomenon is replicated in numerical simulations of 3D solid cells by assigning different elastic moduli to the struts and the nodes to correctly predict the elastic properties of the structure. Despite the intrinsic simplifications, the 3D beam-based modeling provides a simple approach to quickly design new structures based on stiffness-to-weight requirements, whereas the 3D solid models provide additional information on the stress–strain distribution, also highlighting the weakest zones, where failure is likely to occur. The numerical models of large arrays, used to define the scaling law, suggest that the chosen topologies have a mainly stretching-dominated behavior—a hallmark of structurally efficient structures—where the modulus scales linearly with the relative density. As future perspectives, we aim to extend this study to other crystal lattices to create a structure–property library including all the unit cells to help the design of tailored lightweight structures through the implementation of a voxel-based homogenized modeling approach. Lastly, optimization-based design may also represent an exciting path for future work.
