# Supplementary material for: 3D-Printed Architected Materials Inspired by Cubic Bravais Lattices
Source: ACS Biomater Sci Eng. 2021 Jul 26;9(7):3935–44. doi: 10.1021/acsbiomaterials.0c01708 (PMC10336745; doi:10.1021/acsbiomaterials.0c01708)
Supplement: Supplementary file 1 — ab0c01708_si_001.pdf [file ab0c01708_si_001.pdf]

Supporting Information

3D-printed architected materials inspired by cubic Bravais Lattices

Flavia Libonati[1,2], Serena Graziosi[2§], Federico Ballo[2§], Marco Mognato[2], Giacomo Sala[2]

[1] *Department of Mechanical, Energy, Management and Transportation Engineering (DIME)*
Polytechnic School - University of Genoa, Via all'Opera Pia 15/A - 16145 Genova, Italy

[2] *Department of Mechanical Engineering, Politecnico di Milano*
via La Masa 1, 20156 Milano, Italy

[§] *equal contribution*
**Corresponding author. E-mail: flavia.libonati@unige.it*

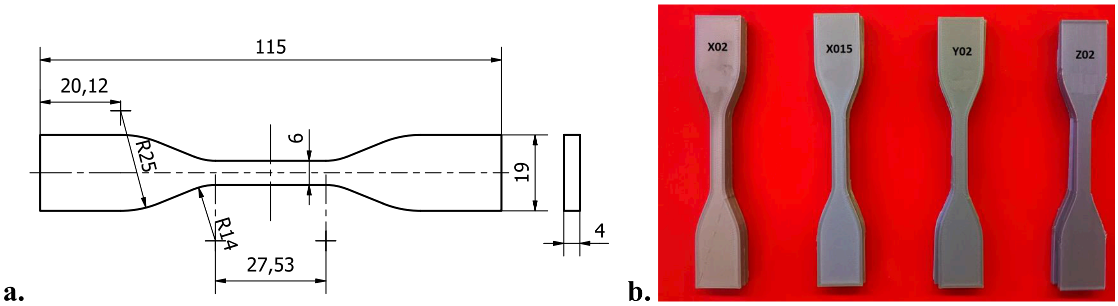

Figure S1 – a) Geometry and dimensions of the dogbone sample (ASTM D638-14). b) 3D-printed samples.

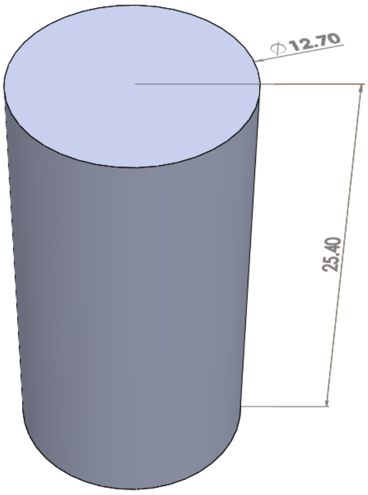

Figure S2 – Geometry and dimensions of the cylindrical sample for compression tests (ASTM-D695).

Table S1 - Calibration of 3D solid models: Elastic moduli applied to nodes and struts for each numerical simulation (for each lattice topology).

Combination	% at Nodes	E at Nodes (MPa)	% at Struts	E at Struts (MPa)
1	100%	3385	100%	3385
2	100%	3385	90%	3046.5
3	100%	3385	80%	2708
4	100%	3385	70%	2369.5
5	100%	3385	60%	2031
6	100%	3385	50%	1692.5
7	90%	3046.5	90%	3046.5
8	90%	3046.5	80%	2708
9	90%	3046.5	70%	2369.5
10	90%	3046.5	60%	2031
11	90%	3046.5	50%	1692.5
12	80%	2708	80%	2708
13	80%	2708	70%	2369.5
14	80%	2708	60%	2031
15	80%	2708	50%	1692.5
16	70%	2369.5	70%	2369.5
17	70%	2369.5	60%	2031
18	70%	2369.5	50%	1692.5
19	60%	2031	60%	2031
20	60%	2031	50%	1692.5
21	50%	1692.5	50%	1692.5

Table S2 – Calibration of 3D beam models: Elastic moduli applied to each numerical simulation (for each lattice topology).

RUN	%	$E(MPa)$
1	100%	3385
2	90%	3047
3	80%	2708
4	70%	2370
5	60%	2031
6	55%	1862
7	50%	1693

Table S3 – 3D solid models: Elastic moduli that best approximate the experimental behavior.

Cell	Elastic Modulus assigned to Nodes [MPa]	Elastic Modulus Assigned to Struts [MPa]
**SC**	2708	2031
**BCC**	3047	2031
**FCC**	2708	2031

Table S4 – 3D beam models: Elastic moduli that best approximate the experimental behavior.

Cell	Elastic Modulus assigned [MPa]
**SC**	1693
**BCC**	1862
**FCC**	2031

Table S5 – Experimental results of the SC cells.

	Max Load $[N]$	Stiffness $[N/mm]$	Elastic modulus $[MPa]$	Max Stress $[MPa]$	Deformation Energy [MJ/m^3]	Max Strain [-]
**Average SC**	437.0 ± 52.0	1136.0 ± 131.2	71.05 ± 8.23	1.71 ± 0.21	0.068 ± 0.018	0.090 ± 0.018
**SC015-L**	451.5 ± 102.1	1290.2 ± 168.1	80.62 ± 10.51	1.76 ± 0.40	0.066 ± 0.032	0.076 ± 0.023
**SC015-T**	443.1 ± 60.4	1034.7 ± 203.5	64.63 ± 12.74	1.73 ± 0.24	0.076 ± 0.021	0.090 ± 0.017
**SC02-L**	430.7 ± 30.3	1292.3 ± 96.7	80.83 ± 6.02	1.68 ± 0.12	0.059 ± 0.008	0.085 ± 0.010
**SC02-T**	422.5 ± 15.1	926.4 ± 56.6	57.93 ± 3.56	1.65 ± 0.06	0.072 ± 0.011	0.108 ± 0.020

Table S6– Experimental results of the BCC cells.

	Max Load $[N]$	Stiffness $[N$/mm]	Elastic modulus $[MPa]$	Max Stress $[MPa]$	Deformation Energy [MJ/m^3]	Max Strain [-]
**Average BCC**	685.5 ± 52.9	1463.0 ± 83.0	91.43 ± 5.19	2.68 ± 0.21	0.140 ± 0.020	0.102 ± 0.014
**BCC015-L**	655.5 ± 82.6	1527.9 ± 158.4	95.49 ± 9.90	2.56 ± 0.32	0.123 ± 0.014	0.099 ± 0.016
**BCC015-T**	771.4 ± 20.4	1529.7 ± 68.9	95.61 ± 4.30	3.01 ± 0.08	0.169 ± 0.013	0.111 ± 0.017
**BCC02-L**	606.5 ± 69.0	1459.2 ± 62.3	91.20 ± 3.89	2.37 ± 0.27	0.107 ± 0.026	0.087 ± 0.014
**BCC02-T**	708.6 ± 39.4	1335.0 ± 42.5	83.33 ± 2.65	2.77 ± 0.15	0.160 ± 0.026	0.111 ± 0.010

Table S7 – Experimental results of the FCC cells.

	Max Load [N]	Stiffness [N/mm]	Elastic modulus [MPa]	Max Stress [MPa]	Deformation Energy [MJ/m^3]	Max Strain [-]
**Average FCC**	1019.0 ± 49.5	2192.8 ± 314.8	139.8 ± 18.4	3.98 ± 0.19	0.276 ± 0.074	0.121 ± 0.025
**FCC015-L**	1007.5 ± 27.8	2526.6 ± 650.0	158.6 ± 38.6	3.94 ± 0.11	0.283 ± 0.180	0.116 ± 0.031
**FCC015-T**	1089.8 ± 64.6	2156.0 ± 120.0	141.0 ± 6.4	4.26 ± 0.25	0.282 ± 0.051	0.105 ± 0.017
**FCC02-L**	927.5 ± 48.1	2041.2 ± 369.1	129.5 ± 22.5	3.62 ± 0.19	0.267 ± 0.016	0.144 ± 0.035
**FCC02-T**	1051.2 ± 57.5	2047.5 ± 120.2	130.2 ± 6.0	4.11 ± 0.22	0.270 ± 0.050	0.119 ± 0.015

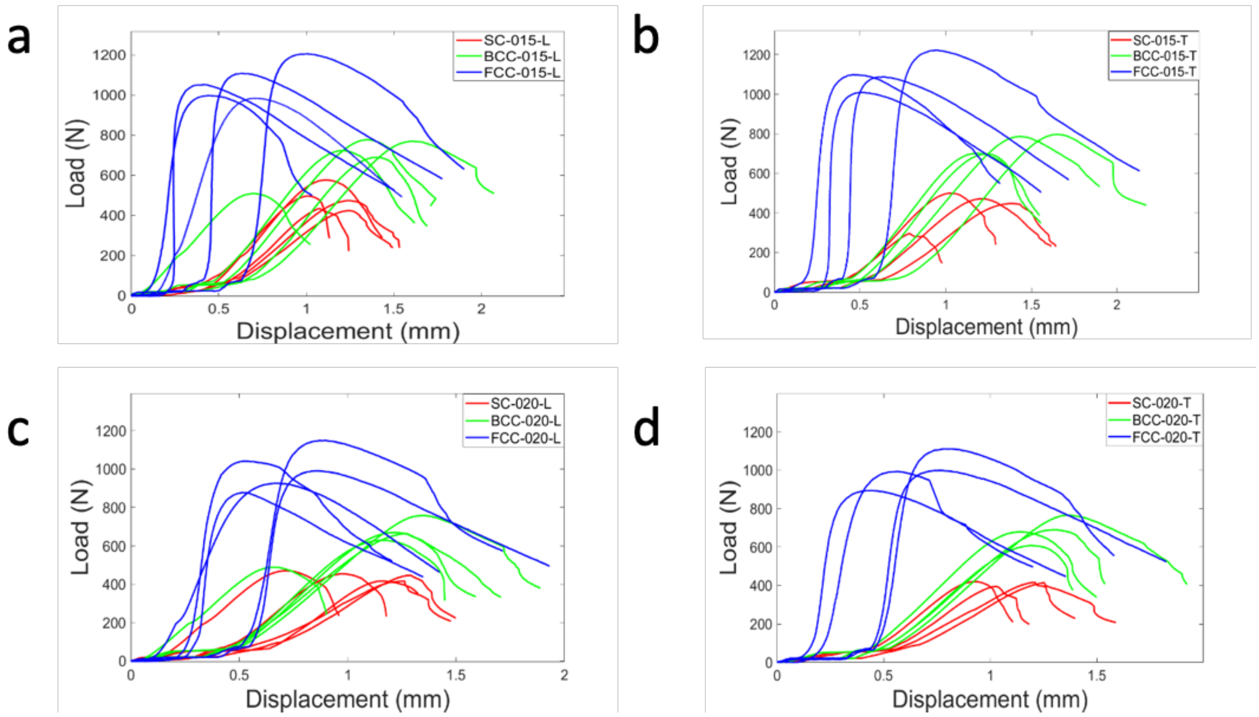

Figure S3 – Load-displacement curves of all the unit cells. The acronym indicates the unit cell, the number indicates the layer thickness and the final letter the loading direction (L, longitudinal and T, transversal).

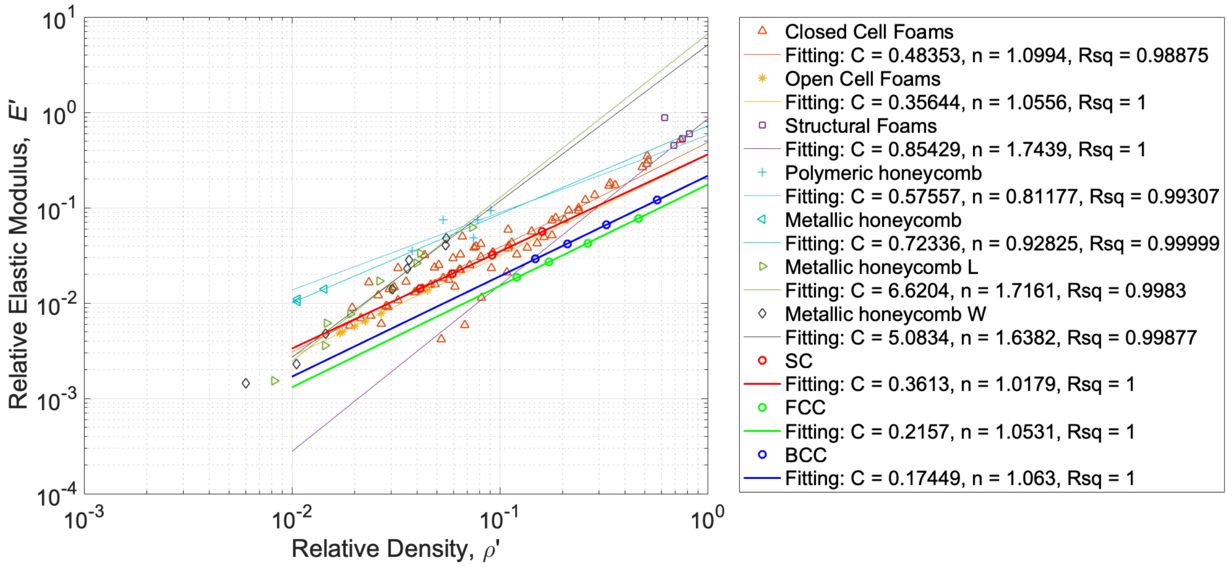

Figure S4 – Ashby plot and scaling laws for different cellular materials (data taken from CES Edu Pack) and comparison with the studied lattices (data taken from FE-simulations).
